# Ultrasound-Guided Injection of Botulinum Toxin Type A for Piriformis Muscle Syndrome: A Case Report and Review of the Literature

**DOI:** 10.3390/toxins7083045

**Published:** 2015-08-10

**Authors:** Andrea Santamato, Maria Francesca Micello, Giovanni Valeno, Raffaele Beatrice, Nicoletta Cinone, Alessio Baricich, Alessandro Picelli, Francesco Panza, Giancarlo Logroscino, Pietro Fiore, Maurizio Ranieri

**Affiliations:** 1 Physical Medicine and Rehabilitation Section, “OORR” Hospital, University of Foggia, Foggia 71100, Italy; E-Mails: francescami@live.it (M.F.M.); gvaleno@libero.it (G.V.); raffaelebeatrice@tiscali.it (R.B.); n.cinone@gmail.com (N.C.); maurizio.ranieri@unifg.it (M.R.); 2 Physical Medicine and Rehabilitation, Department of Health Sciences, University of Eastern Piedmont, Novara 28100, Italy; E-Mail: alessio.bar@gmail.com; 3 Neuromotor and Cognitive Rehabilitation Research Center, Department of Neurological, Biomedical and Movement Sciences, University of Verona, Verona 37134, Italy; E-Mail: alessandro.picelli@univr.it; 4 Neurodegenerative Disease Unit, Department of Basic Medicine, Neuroscience, and Sense Organs, University of Bari Aldo Moro, Bari 70120, Italy; E-Mails: geriat.dot@geriatria.uniba.it (F.P.); giancarlo.logroscino@uniba.it (G.L.); 5 Department of Clinical Research in Neurology, University of Bari Aldo Moro, “Pia Fondazione Cardinale G. Panico”, Tricase, Lecce 73039, Italy; 6 Geriatric Unit & Laboratory of Gerontology and Geriatrics, Department of Medical Sciences, IRCCS “Casa Sollievo della Sofferenza”, San Giovanni Rotondo, Foggia 71013, Italy; 7 Department of Basic Medicine, Neuroscience, and Sense Organs, University of Bari Aldo Moro, Bari 70120, Italy; E-Mail: pietro_fiore@hotmail.it

**Keywords:** piriformis muscle syndrome, botulinum toxin type A, ultrasound guide

## Abstract

Piriformis muscle syndrome (PMS) is caused by prolonged or excessive contraction of the piriformis muscle associated with pain in the buttocks, hips, and lower limbs because of the close proximity to the sciatic nerve. Botulinum toxin type A (BoNT-A) reduces muscle hypertonia as well as muscle contracture and pain inhibiting substance P release and other inflammatory factors. BoNT-A injection technique is important considering the difficult access of the needle for deep location, the small size of the muscle, and the proximity to neurovascular structures. Ultrasound guidance is easy to use and painless and several studies describe its use during BoNT-A administration in PMS. In the present review article, we briefly updated current knowledge regarding the BoNT therapy of PMS, describing also a case report in which this syndrome was treated with an ultrasound-guided injection of incobotulinumtoxin A. Pain reduction with an increase of hip articular range of motion in this patient with PMS confirmed the effectiveness of BoNT-A injection for the management of this syndrome.

## 1. Introduction

The term “piriformis muscle syndrome” (PMS) identifies a condition of buttocks and posterior hip pain with limited articular range of motion (ROM) of the hip not caused by lumbar radiculopathies, but produced by prolonged or excessive contraction of the piriformis muscle. Subjects suffering from this syndrome refer a sciatic-like pain. This can be explained considering that proximal sciatic nerve can be compressed by the anatomical variations of the piriformis muscle itself or can be injured by the scar tissue of the piriformis muscle and adjacent tissues from trauma of the gluteal region or for pinching of sciatic nerve by the piriformis muscle during leg and hip maneuvers [1]. Several therapies can be used for pain relief with controversial results. Conservative strategies include hip extensor, abductor, and adductor muscle stretching exercises, massage, physical therapies (heat, ultrasound, laser therapy), and drug treatments (nonsteroidal anti-inflammatory drugs, analgesics, and muscle relaxants). If the conservative regimen fails, local injection of piriformis muscle, surgery and trigger-point injections with local anesthetic with or without corticosteroids should be performed into piriformis muscle [2]. In recent years, several published studies showed the employment of botulinum toxin type A (BoNT-A) injection as a new therapeutic option to reduce buttocks and low back pain induced by PMS [3,4,5,6]. Botulinum toxin is actually the gold standard therapy in the management of focal hypertonia by blocking the release of acetylcholine at the neuromuscular junctions in the injected muscle and resulting in a reduction of spasticity, dystonia, and related disorders [7,8]. Moreover, BoNT-A has been shown to reduce muscle pain with safety and effectiveness [9,10,11,12]. Muscle pain can result from ischemic, thermal, or mechanical stimulation and it is usually described as aching or cramping and it is often difficult to precisely localize. The list of factors known to sensitize muscular nociceptors includes bradykinins, serotonin, potassium, prostaglandin E2 and a variety of neuropeptides, such as substance P and the neuropeptide calcitonin gene-related peptide (CGRP). BoNT-A has been found to inhibit substance P release from cultured embryonic dorsal root ganglion neurons and to reduce stimulated (but not basal) release of CGRP from cultured trigeminal ganglia neurons. Based on these results, BoNT-A may inhibit the release of these neuropeptides *in vivo*, which may account for its beneficial effects on pain [13,14,15]. Moreover, the concentrations and release patterns of the sensitizing mediators may respond directly to the reduction of excessive contractions or spasms evoked by botulinum toxin reducing also the increase of lactate in the contracted muscle causing of muscle pain. In the present review article, we briefly reviewed current knowledge about the employment of BoNT-A for the treatment of PMS, also describing a case report about its use with an ultrasound-guided injection technique for the management of this syndrome.

## 2. Methods

In the present article, we reviewed existing literature about the use of BoNT-A in the treatment of PMS, including randomized placebo-controlled studies, double-blind trials, case-reports, and meta-analyses published in the period from December 1989 to July 2014. We chose this starting date for our literature search because in December 1989, BoNT-A was approved by the US Food and Drug Administration for the treatment of strabismus, blepharospasm, and hemifacial spasm in patients over 12 years old. This narrative review was based upon searches of the US National Library of Medicine (PubMed), Ovid MEDLINE, EMBASE, Google Scholar, Web of Science, and Scopus databases using combinations of the following terms to identify the type of treatment (piriformis muscle syndrome OR PMS and botulinum toxin AND piriformis muscle syndrome OR PMS treated with botulinum toxin) combined with terms to determine the area of interest (muscle pain OR myofascial pain OR myofascial pain syndrome). A search filter was developed to include only human studies. There were no language restrictions on the search. The references of each study selected were screened to identify studies that were not included on the electronic search. Key textbooks were also searched in addition to the electronic database search. References mentioned in the textbooks were likewise reviewed. From 32 articles identified with multiple electronic searches, we screened titles and abstracts of the citations downloaded from the searches identifying 16 potential relevant articles chosen for a closer review. Excluding the other five articles that did not meet the inclusion criteria, we obtained full copies of the 11 potentially suitable reports for further assessment. After inclusion of one article of interest from the reference lists of the selected articles and exclusion of other three articles, nine studies met study eligibility criteria, and were finally included in the overall review [3,4,6,16,17,18,19,20,21] (Table 1).

**Table 1:** Key and reviewed studies on the employment of botulinum toxin type A (BoNT-A) and type B (BoNT-B) therapy in the treatment of piriformis muscle syndrome (PMS).

First author and year of publication	Study design	Number of patients with PMS	Doses (U) and number of injections	Injection guide	Outcome measures	Adjunctive therapy	Clinical results/adverse effects
Fanucci *et al.*, 2001 [4]	Prospective	30	OnabotulinumtoxinA 100 U: 26 received one injections 4 received two injections	CT	Not specific pain score	Not indicated	Reduction of pain intensity after 5–7 days for 26 subjects; other 4 subjects reported a complete pain reduction in the following week/No adverse effects
Fishman *et al.*, 2002 [17]	RCT double-blind, parallel	21	AbobotulinumtoxinA 200 U	EMG	VAS	Twice-weekly physical therapy program	65% of the patients reported a 50% of pain reduction/No adverse effects
Childers *et al.*, 2002 [3]	RCT double-blind, crossover	9	OnabotulinumtoxinA 100 U	FL EMG	VAS	Not indicated	Reduction of pain intensity, distress, spasm and interference with activities respect to baseline/No adverse effects
Fishman *et al.*, 2004 [20]	Prospective	27	BoNT-B 12,500 U	EMG	VAS	Physical therapy twice weekly for 3 months	A total of 24 of 27 study patients had a pain relief of 50%. The most severe adverse effects were dry mouth and dysphagia, approaching 50% of patients at 2 and 4 weeks
Lang *et al.*, 2004 [21]	Prospective	20	BoNT-B 5,000 U	EMG	VAS	Dry mouth was reported in 6 of 20 patients	Reduction of myofascial buttock and hip pain associated with PMS/No adverse effects
Yoon *et al.*, 2006 [6]	Prospective	20	AbobotulinumtoxinA 150 U: One injection	CT	NRS at 4, 8, 12 weeks Korean SF-36	Stretching exercise 20 times/day for 12 week	Pain reduction respect to baseline(*p* < 0.0001) Improvement of physical functioning, role physical, bodily pain, general health, vitality and social functioning/Mild and transient adverse events (one case of flu-like syndrome lasting 2 days, five cases of worsening muscular pain lasting 2–3 days, one patient had ecchymosis in the lower limb for 2 days, two patients had atrophy of the piriformis muscle, one case of transient numbness lasting no longer than 72 h
Michel *et al.*, 2013 [16]	Prospective	122	OnabotulinumtoxinA Ranged between 50 and 100 U: 51 received one Injection; 43 received two injections; 18 received three injections; 9 received four injections; 1 received five injections	EMG	VAS	Not indicated	“Very good’ or ‘Good” in 77% of the cases “Average” in 7.4% of the cases “Poor” in 15.6% of the cases/No adverse effects
Fabregat *et al.*, 2014 [18]	Feasibility study	10	OnabotulinumtoxinA 100 U	US	Not indicated	Not indicated	No adverse effects during the BoNT-A injections
Al-Al-Shaikh *et al.*, 2015 [19]	Retrospective	12	OnabotulinumtoxinA 100 U	US CT	VAS	Not indicated	Buttock and sciatic pain reduction respect to baseline (*p* < 0.001)/No adverse effects

CT: Computed Tomography; RCT: Randomized Clinical Trial; EMG: Electromyography; VAS: Visual Analogue Scale; FL: Fluoroscopy; NRS: Numeric Rating Scale; SF-36: 36-Item Short Form Health Survey; US: Ultrasound.

## 3. Botulinum Toxin in Piriformis Muscle Syndrome

Many studies described the use of botulinum toxin for reducing muscle and myofascial pain, but there are only few reports about the treatment of PMS [3,4,6,16,17,18,19,20,21] (Table 1). One of the first studies showing the use of BoNT-A was conducted by Fanucci and colleagues on thirty subjects suffering from PMS and treated with onabotulinumtoxin A [4]. The authors referred to have treated with 100 units (U)/1 mL of saline, under computed tomographic (CT) guidance using a 20 G needle. No complications or adverse reaction were reported. Among the treated subjects, 26 subjects referred a gradual relief of symptoms after 5–7 days, whereas 4 subjects needed another CT-guided injection of 100 U/2 mL onabotulinumtoxin A resulting a complete pain reduction in the following week [4]. The efficacy of onabotulinumtoxin A 100 U compared to saline solution was examined in a double-blind, single group study. Nine women suffering from PMS received fluoroscopic/electromyography (EMG)-guided piriformis injections and visual analogue scale (VAS) was used to measure pain intensity, distress, spasm, and interference with activities. After injection with placebo, decreases of these symptoms were detected, but only in one of the four categories (distress) whereas after injection with BoNT-A decreases were observed under all VAS categories suggesting that 100 U of onabotulinumtoxin A can reduce pain to a greater extent than similar injections with saline solution alone [3].

Fishman and colleagues compared the efficacy of abobotulinumtoxin A 200 U (21 subjects), triamcinolone and lidocaine (31 subjects), and normal saline as placebo (15 subjects) used in conjunction with physical therapy for the treatment of PMS [17]. A 50% reduction in VAS scores was used as the main outcome. As measured on the VAS, patients injected with abobotulinumtoxin A (65%) experienced more relief from pain than patients receiving lidocaine with steroid (32%) and more relief than patients receiving placebo (6%). Authors concluded that treatment with abobotulinumtoxin A for PMS provided therapeutic effectiveness that is superior to steroid and to placebo [17].

In a prospective, single-site, open-label trial, 20 subjects with PMS were treated with 150 U of abobotulinumtoxin A in 3 mL of normal saline into the affected unilateral piriformis muscle using a CT-guidance [6]. After the injection each patient was given instructions to perform standard stretching exercises 20 times each day for 12 weeks after treatment. Outcome measures included a numeric rating scale for pain at baseline and at 4, 8, and 12 weeks after treatment. The Korean version of the 36-Item Short Form Health Survey (SF-36) was used to evaluate the health-related quality of life at baseline and at 4 weeks of treatment. The patients reported an important reduction of pain with an improvement of quality life in terms of physical functioning, role physical, bodily pain, general health, vitality, and social functioning [6]. Only mild and transient adverse events were reported (one case of flu-like syndrome lasting two days, five cases of worsening muscular pain lasting 2–3 days, one patient had ecchymosis in the lower limb for two days, two patients had atrophy of the piriformis muscle, one case of transient numbness lasting no longer than 72 h) [6].

Michel and colleagues demonstrated the efficacy of BoNT-A administration into piriformis muscle in 122 subjects who were previously treated with medication and rehabilitation protocols with no pain improvement [16]. A dose of onabotulinumtoxin A between 50 and 100 U was injected under guidance with EMG. The minimum interval between injections was 3 months. A group of 51 patients received one single injection, 43 subjects had two injections, 18 had three injections, nine had four injections, and only one had five injections. The average interval was 18 weeks between the first and second injections, 31 weeks between second and third injections, 45 weeks between third and fourth injections, and 57 weeks between fourth and the fifth injections. The patients injected have been evaluated using the VAS: Pain relief was ‘very good’ or ‘good’ in 77% of the cases, ‘average’ in 7.4% and ‘poor’ in 15.6% of the cases. No adverse events have been reported.

The important role of instrumental guidance to inject piriformis muscle has been described also by Fabregat and colleagues [18] that demonstrated the effectiveness of ultrasound (US)-guided procedure to identify the muscle injecting 10 patients suffering from PMS with 100 U of onabotulinumtoxin A reconstituted with normal saline for a total volume of 5 mL [18]. Furthermore, in a single-centre, case-control study conducted by Al-Al-Shaikh and colleagues, the authors described the changes in the piriformis muscle morphology induced by onabotulinumtoxin A 100 U administered into the piriformis muscle (10 subjects) under US guide and into piriformis muscle and obturator internus muscles (2 subjects) under CT guidance. The authors reported a significant difference on VAS in terms of buttock pain and sciatic pain before and after treatment [19]. Finally, another serotype of botulinum toxin can be used to reduce muscle pain. In fact, a study showed the safety and the effectiveness of physical therapy and 12,500 units of BoNT-B administered under EMG-guidance in 27 subjects suffering from PMS, for more than 3 months [20], whereas Lang evaluated the clinical safety and efficacy of 5000 U of BoNT-B under EMG-guidance in 20 patients with PMS, demonstrating a reduction of myofascial pain associated with this syndrome [21]. In these studies with BoNT-B in PMS, the most severe adverse effects were dry mouth and dysphagia, approaching 50% of patients at two and four weeks in the first report [20] and dry mouth reported in six of 20 patients in the report of Lang [21].

## 4. Case Report

In this report, we described the case of a 55-years-old man with right buttock and leg pain exacerbated by the sitting position and hip extra-rotation. Pain affected everyday activities including his job. In fact, the patient was a driver and he needed to spend much time sitting. Physiatric examination revealed no neurologic signs of lumbar herniatic disc or spinal cord compression. These clinical findings were confirmed by instrumental diagnostic investigations. The magnetic resonance imaging (MRI) did not show any vertebral alterations and no neurogenic muscle atrophy or sciatic and femoral nerves damage was detected by EMG. Hip radiographs excluded any orthopedic cause of pain. A trigger point was identified by palpation of the right buttock with pain radiating down the thigh. Considering these symptoms, a PMS was suspected. Specific tests were performed to validate the diagnosis. In particular, the FAIR test was applied downward pressure to the symptomatic flexed knee while maximizing the adduction and internal rotation in the symptomatic flexed hip in the lateral decubitus position [22], and Freiberg’s maneuver was performed with patient in supine position and rotating internally the extended thigh on the affected side [23]. Finally, Pace’s maneuver, actively abducting both thighs against resistance in the seated position was done [24]. All these tests were positive. A US-guided injection of local anesthetic into the piriformis muscle was performed. The marked and immediate relief of pain confirmed the diagnosis of PMS [25].

The active articular ROM of the coxo-femoral joint was limited by pain with an extra-rotation of 20°, abduction of 25° and extension of 10°. Pain score was measured using the VAS [26]. VAS score in sitting position was 9/10. The patient was initially treated with oral drugs (muscle relaxant and anti-inflammatory medications, benzodiazepines), physical therapies (high-intensity laser therapy, diatermia, and US therapy), hip and thigh mobilization, gluteus muscles massages in order to reduce piriformis muscle spasm and contracture. No benefits were observed after these treatments. A botulinum neurotoxin therapy was then started. This decision was taken according to previous studies [3,4,6,16,17,18,19,20,21] (Table 1). 

**Figure 1:**
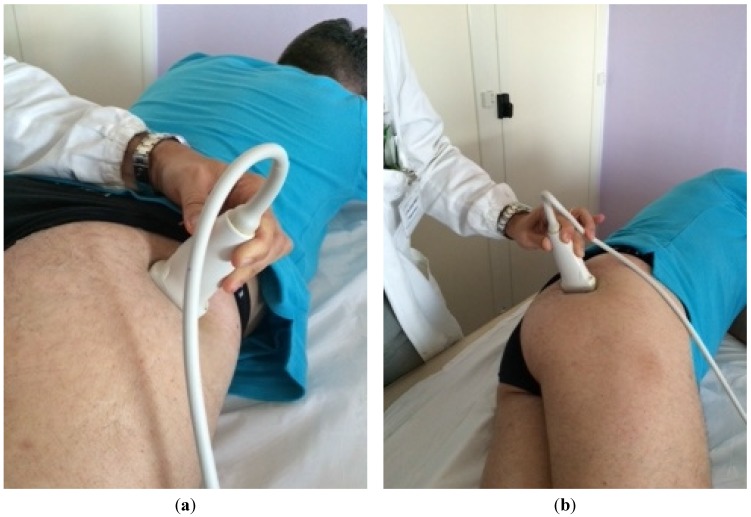
Positioning of the probe initially with its lateral side medial to the greater trochanter (**a**) and finally positioned with its medial side lateral to the ischial tuberosity (**b**).

**Figure 2:**
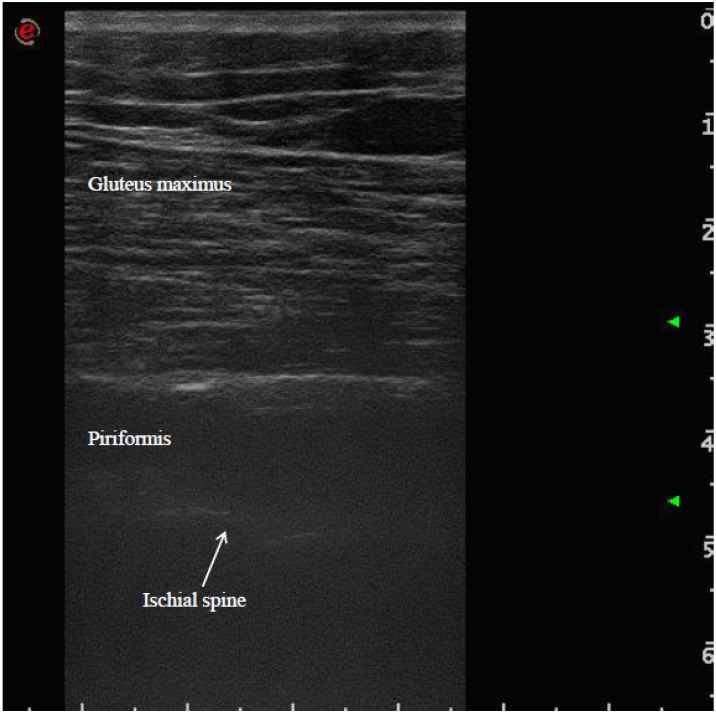
Ultrasound identification of the piriformis muscle between gluteus maximus muscle and ischial spine.

The patient was placed in a prone position. The method of locating the sciatic nerve as proposed by Smith and colleagues was adopted [27]. The B-mode real-time ultrasonography was performed using the MyLab 70 XV (Esaote, Genova, Italy) with a linear transducer (scanning frequency, 6–18 MHz) to guide the needle positioning into the targeted muscle. The transducer was positioned in the transverse view, perpendicular to the gluteus maximus muscle. The probe was initially positioned with its lateral side medial to the greater trochanter and then with its medial side lateral to the ischial tuberosity (Figure 1). The piriformis muscle was identified between gluteal muscles and ischial spine (Figure 2). The patient was treated with incobotulinumtoxin A, using a 22 gauge (0.70 × 90 mm) spinal needle, injecting the right piriformis muscle into one point with a dose of 40 U with 2 cc of saline dilution. The procedure was well tolerated and no adverse effects were noted. No additional treatments were performed. 

A few days after the treatment, the patient reported a significant regression of most signs and symptoms, in terms of pain (VAS score: 2/10) and functional outcome: The range of hip external rotation was significantly higher and ranged to 45°, as well as abduction and extension movements, respectively 40° and 20*°.* Quality of life and work also improved, the patient was able to spend more time sitting and could easily get in and out of the car; extra rotating his hip with no pain. In the eighth month of follow up, the positive effect of BoNT-A injection into piriformis muscle was still present.

## 5. Discussion

In the present report, we described a case of PMS treated with BoNT-A in a 55-years-old man who referred a right buttock and leg pain exacerbated with sitting position and hip extra-rotation. The absence of radicular signs for lumbar hernia or spinal cord compression as well as the reduction of pain after a local piriformis muscle anesthetic injection under US guidance, confirmed the diagnosis. 

PMS is a common cause of muscle pain secondary to piriformis contracture with sciatic nerve impairment. As described by Stewart, four pathological conditions can be identified: damage to the proximal sciatic nerve by lesions in the vicinity of the piriformis; compression damage to the proximal sciatic nerve by the piriformis; damage to the sciatic nerve by the piriformis; and adjacent tissues from trauma and scarring and chronic buttock pain with no evidence of sciatic nerve damage [28]. BoNT injections have been used to treat muscle and myofascial pain in several studies [10,11,12]. Muscle pain in PMS can be evoked by a chemical C and A fibers stimulation because of local production of lactate and acid PH induced by long-term contracture and ischemia related. The reduction of muscle contracture obtained by BoNT-A or BoNT-B injections may explain the pain decrease. Moreover, BoNT-A has been found to inhibit bradykinins, serotonin, potassium, prostaglandin E2, substance P and the neuropeptide CGRP reducing sensitization of muscle nociceptors [13,14,15]. For this reason, BoNT-A can be used, as well as steroid methylprednisolone with bupivacaine, to treat myofascial pain syndrome and pain from chronic muscle spasm [29]. 

However, treatment effectiveness is strictly related to injection accuracy, reducing also, especially using anesthetic drug, the risk of sciatic nerve sensorimotor block. In fact considering the difficult access of the needle for deep location, the small size of the muscle, the proximity to neurovascular structures, and the possibility of anomalous sciatic nerve and piriformis arrangement, several techniques of piriformis injections have been proposed and described: CT, MRI, US, fluoroscopy, electrical stimulators, or EMG [3,4,6,16,17,18,19,20,21,30,31,32,33,34,35,36]. Nerve stimulator and anatomic landmarks have been proposed by Hanania and colleagues [30,31]. Fishman and colleagues described a technique using EMG to confirm a correct needle placement correlating with the fluoroscopic image [32]. Betts described the use electrical muscle stimulation to better define the location of the piriformis muscle and the fluoroscopy helped to ensure precise needle placement for an accurate injection [33], whereas Gonzalez and colleagues did not use electrical stimulation but anatomic landmarks and fluoroscopic guidance to document the needle placement within the piriformis muscle of a cadaveric specimen [34]. Ultrasonography is well established as a reliable and reproducible imaging method in muscle anatomy, and several studies have shown applicability of the procedure for visually-controlled BoNT-A injection [35]. Smith and colleagues confirmed the advantages of US-guided piriformis injection technique, considering accessibility, compact size, lack of ionizing radiation exposure, and direct visualization of neurovascular structures [27]. In conclusion, the findings of the present case report are similar to those of previous studies on this topic [3,4,6,16,17,18,19,20,21] (Table 1). After incobotulinumtoxin A injection, the buttock and posterior hip pain was drastically reduced improving hip functionality and quality of work and life. BoNT-A may be an agent suitable for PMS. On the other hand, a recent Cochrane review, found that BoNT injections were better than injections of corticosteroid plus lidocaine, or traditional acupuncture or placebo in patients with sciatica attributed to PMS [36]. Instrumental guidance of injections is necessary to avoid the BoNT spreading to neurovascular structures. In the future, careful blinded US-guided studies in a larger number of patients are needed to establish the efficacy of BoNT injections in PMS.
